# Overweight and obesity among children and adolescents in Germany. Results of the cross-sectional KiGGS Wave 2 study and trends

**DOI:** 10.17886/RKI-GBE-2018-022.2

**Published:** 2018-03-15

**Authors:** Anja Schienkiewitz, Anna-Kristin Brettschneider, Stefan Damerow, Angelika Schaffrath Rosario

**Affiliations:** Robert Koch Institute, Berlin, Department of Epidemiology and Health Monitoring

**Keywords:** OVERWEIGHT, OBESITY, EXAMINATION SURVEY, HEALTH MONITORING, KIGGS

## Abstract

For some time, there have been indications that the prevalence of overweight and obesity among children and adolescents in Germany has stabilised at a high level. The second wave of the German Health Interview and Examination Survey for Children and Adolescents (KiGGS Wave 2, 2014-2017) once again provides nationwide measurements on height and weight of children and adolescents aged 3 to 17 years. The results are confirming this trend. The prevalence of overweight is 15.4% and 5.9% for obesity. There are no differences between girls and boys. Overweight and obesity prevalence increases with age. Children and adolescents with low socioeconomic status (SES) are more likely to be overweight and obese than those with high SES. Compared to the KiGGS baseline study (2003-2006), there was no further increase in overweight and obesity prevalence overall and in all age groups.

## Background

The high prevalence of overweight and obesity in childhood and adolescence is a global health problem and a major public health challenge in the 21st century. The prevention of excessive weight gain in children and adolescents has a high relevance for various reasons: Children with overweight and obesity are more likely to suffer from cardiovascular risk factors such as high blood pressure, as well as disorders in lipid and in glucose metabolism, when compared to their normal-weight peers [1]. In addition, a high body mass index (BMI) in childhood and adolescence is associated with a higher likelihood of type 2 diabetes, hypertension and cardiovascular disease in adulthood [2]. Furthermore, overweight and obesity in children and adolescents are associated with a significant reduction in quality of life [3] and a higher risk of bullying [4].

Since the mid-1970s, an increase in the prevalence of overweight and obesity among children and adolescents has been observed worldwide [5]. However, since the beginning of the 2000s, it has become apparent for many high-income countries that the trend of increasing overweight and obesity prevalences is not continuing [6, 7]. There are also indications for Germany that the prevalences are not increasing, or that the trend is slowing down, or even levelling off [8-13].

According to the results of the German Health Interview and Examination Survey for Children and Adolescents (KiGGS baseline study), which was conducted between 2003 and 2006, a total of 15% of children and adolescents aged 3 to 17 years were either overweight or obese. Obesity was observed in 6.3% of children and adolescents [14]. From the first follow-up survey (KiGGS Wave 1), which took place between 2009 and 2012, self-reported data on body height and weight were available, complemented by measured values from a subsample. In order to compare the self-reported data with the measured values of the KiGGS baseline study, the self-reported data were adjusted using a correction formula. The prevalence of overweight and obesity among children and adolescents had not increased further, but they were still at a high level [9, 10].

**KiGGS Wave 2**

Second follow-up to the German Health Interview and Examination Survey for Children and Adolescents

**Data owner:** Robert Koch Institute

**Aim:** Providing reliable information on health status, health-related behaviour, living conditions, protective and risk factors, and health care among children, adolescents and young adults living in Germany, with the possibility of trend and longitudinal analyses

**Study design**: Combined cross-sectional and cohort study

**Cross-sectional study in KiGGS Wave 2**

**Age range:** 0-17 years

**Population:** Children and adolescents with permanent residence in Germany

**Sampling:** Samples from official residency registries - randomly selected children and adolescents from the 167 cities and municipalities covered by the KiGGS baseline study

**Sample size:** 15,023 participants

**KiGGS cohort study in KiGGS Wave 2**

**Age range:** 10-31 years

**Sampling:** Re-invitation of everyone who took part in the KiGGS baseline study and who was willing to participate in a follow-up

**Sample size:** 10,853 participants

**KiGGS survey waves**

► KiGGS baseline study (2003-2006), examination and interview survey
► KiGGS Wave 1 (2009-2012), interview survey
► KiGGS Wave 2 (2014-2017), examination and interview survey

More information is available at www.kiggs-studie.de/english

With KiGGS Wave 2, which was conducted between 2014 and 2017, measurements on height and weight of children and adolescents aged 3 to 17 years living in Germany are now available again. Thus, the most recent national population-based estimates of overweight and obesity can be provided and the development since the last survey eleven years ago can be reported.

## Indicator and methodology

KiGGS is part of the health monitoring system undertaken at the Robert Koch Institute. It includes repeated cross-sectional surveys that are representative for children and adolescents aged between 0 and 17 years in the German population (KiGGS cross-sectional study). After conducting the baseline study as an interview and examination survey between 2003 and 2006, and KiGGS Wave 1 as an interview-based survey between 2009 and 2012, KiGGS Wave 2 was conducted between 2014 and 2017 as a combined interview and examination survey.

A detailed description of the methodology used in KiGGS Wave 2 can be found in New data for action. Data collection for KiGGS Wave 2 has been completed in issue S3/2017 as well as KiGGS Wave 2 cross-sectional study – participant acquisition, response rates and representativeness in issue 1/2018 of the Journal of Health Monitoring [15, 16].

In the physical examination component of KiGGS Wave 2, standardised measurements of body height and weight of participants aged 3 to 17 years were obtained. The body mass index (BMI, kg/m^2^) was calculated from body weight divided by the square of body height. Since the relationship between body height and weight changes during childhood and adolescence due to growth, there is no uniform cutpoint for all age groups from which a child or adolescent is classified as overweight or obese. For this reason, up to the age of 18 years, BMI percentile curves from a reference population, taking age and gender into account, are used to classify an individual BMI value. In Germany, overweight and obesity are based on the national reference percentiles according to Kromeyer-Hauschild [17, 18]. Children and adolescents are defined as overweight if their BMI is above the 90^th^ age- and gender-specific percentile. A BMI above the 97^th^ percentile is defined as obesity.

The analyses are based on data from 3,561 participants (1,799 girls and 1,762 boys) aged 3 to 17 years with valid measurements of height and weight. The results are presented as prevalences (frequencies) stratified by gender, age and socioeconomic status (SES) [19].

In the calculations a weighting factor was used to correct for deviations of the sample from the German population with regard to age, gender, federal state, German nationality as well as the distribution of parental levels of education (Microcensus 2013 [20]).

This article reports the prevalences with 95% confidence intervals (95%CI). The calculation of trends between the KiGGS baseline study and KiGGS Wave 2 is based on age-standardised prevalence for both survey points and differences were tested through univariable logistic regression. Differences between groups are interpreted as statistically significant if the calculated p-value is smaller than 0.05 taking weighting factor and survey design into account.

## Results and discussion

In KiGGS Wave 2, the prevalence of overweight (including obese) girls and boys aged 3 to 17 years is 15.4%. The prevalence of obesity is 5.9%. There are no statistically significant gender differences. Overweight and obesity prevalences increase with age. The proportion of overweight children is 10.8% for 3 to 6 year old girls and 7.3% for boys. It rises to 16.2% for girls aged 14 to 17 years and 18.5% for boys in this age group. Children and adolescents with low SES have a higher prevalence of overweight than girls and boys with medium and high SES (Table 1).

The obesity prevalence among 3 to 6 year old girls is 3.2%, and 1.0% among boys. This proportion rises to 7.7% for girls aged 14 to 17 years and 9.2% for boys (Table 2). Children and adolescents with low SES are considerably more often affected by obesity: Girls and boys with low SES are about four times as often affected by obesity as children and adolescents with high SES (girls 8.1% vs. 2.0%; boys 11.4% vs. 2.6%). However, this result is only statistically significant among boys.

**Figure 1:**
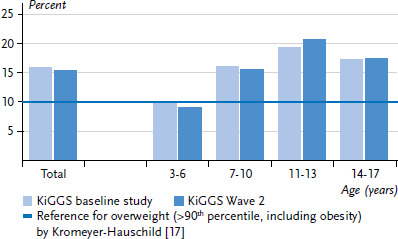
Trend for overweight prevalence (>90^th^ percentile, including obesity) by age group (KiGGS baseline study n=7,215 girls, n=7,531 boys, KiGGS Wave 2 n=1,799 girls, n=1,762 boys) Source: KiGGS baseline study (2003-2006), KiGGS Wave 2 (2014-2017)

**Figure 2:**
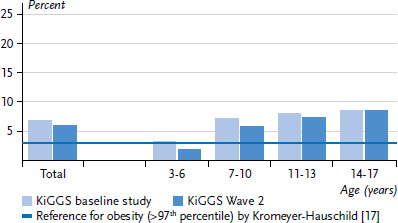
Trend for obesity prevalence (>97^th^ percentile) by age group (KiGGS baseline study n=7,215 girls, n=7,531 boys, KiGGS Wave 2 n=1,799 girls, n=1,762 boys)) Source: KiGGS baseline study (2003-2006), KiGGS Wave 2 (2014-2017)

The results from KiGGS Wave 2 indicate that the increase in overweight and obesity prevalences observed in the KiGGS baseline study in comparison to the reference population has not continued (Figure 1 and Figure 2). Compared to the 1990s reference percentiles, according to which, by definition, 10% of children and adolescents were considered to be overweight (BMI >90^th^ percentile), the results of the KiGGS baseline study showed that the prevalence of overweight (including obesity) in the population had risen to 15%. The prevalence of obesity, by definition 3% of the reference population (BMI >97^th^ percentile), had even doubled to 6% [14]. Since the survey 2003-2006, overweight and obesity prevalences have remained stable overall and across all age groups, albeit at a high level.

These findings are in line with the results of other national studies: although data from school entry health examinations in the federal states showed an increase until 2004, there was no overall increase in the prevalence of overweight and obesity among children at school entry age between 2004 and 2008. However, overweight and obesity prevalences vary widely between federal states [12, 13]. Measurements of body height and weight of children and adolescents between the ages of 4 and 16 years, carried out in paediatric practices and other health centres, also showed a decrease or stabilisation in the prevalence of overweight and obesity [8, 11].

Whether the increase in overweight and obesity prevalence has actually stopped, levelled off or even been reversed is being discussed extensively in science. Aside from methodological factors [21], population-wide interventions and prevention could have led to a stagnation of prevalences over time. In its report “Ending Childhood Obesity”, the World Health Organization (WHO) describes overweight and obesity in childhood and adolescence as a ‘complex and multi-dimensional problem’. Preventive actions for changing individual behavior thus only lead to a limited solution to the problem. Rather, approaches to change the living environment such as altering an increasingly overweight and obesity-promoting (“obesogenic”) environment, should be implemented and considered as a task for the whole of society [22]. Overweight and obesity prevalence among children and adolescents in Germany has not increased further over the last decade. The objective of the WHO’s Global Action Plan for the Prevention and Control of Non-Communicable Diseases to “halt the rise in obesity” by 2025 has thus been achieved. This also applies to the goal of the federal government’s National Sustainable Development Strategy 2016, which is to ensure that the proportion of young people with obesity in Germany does not increase further by 2030 [23, 24]. Nevertheless, the prevalence of overweight and obesity remain at a high level. Against this background, health promotion and prevention activities that contribute to the reduction of overweight and obesity prevalences in the population must continue.

**Erratum, page 18**

In the original article, the number of cases of the KiGGS baseline study and KiGGS Wave 2 were switched in the titles of Figure 1 and Figure 2 on page 18. The number of cases was corrected for the current version in both figure titles: KiGGS baseline study n=7.215 girls, n=7.531 boys; KiGGS Wave 2 n=1.799 girls, n=1.762 boys.

## Key statements

- KiGGS Wave 2 (2014-2017) once again provides measurements on height and weight of 3 to 17 year-old children and adolescents living in Germany.
- The prevalence of overweight (including obesity) in girls and boys aged 3 to 17 years is 15.4% and the prevalence of obesity is 5.9%.
- Participants with a low socioeconomic status are significantly more likely to be overweight than adolescents in the highest status group.
- In comparison to the KiGGS baseline study (2003-2006), there was no further increase in the prevalence of overweight and obesity.

## Figures and Tables

**Table 1:** Prevalence of overweight (>90^th^ percentile, including obesity) according to gender, age and socioeconomic status (n=1,799 girls, n=1,762 boys) Source: KiGGS Wave 2 (2014-2017)

Girls	%	(95% CI)	Boys	%	(95% CI)
**Girls (total)**	**15.3**	**(13.1-17.8)**	**Boys (total)**	**15.6**	**(13.0-18.6)**
**Age**			**Age**		
3-6 Years	10.8	(7.0-16.5)	3-6 Years	7.3	(4.7-11.1)
7-10 Years	14.9	(10.9-20.2)	7-10 Years	16.1	(11.7-21.8)
11-13 Years	20.0	(15.0-26.2)	11-13 Years	21.1	(15.5-28.1)
14-17 Years	16.2	(12.6-20.7)	14-17 Years	18.5	(14.2-23.8)
**Socioeconomic status**			**Socioeconomic status**		
Low	27.0	(20.3-34.9)	Low	24.2	(17.7-32.3)
Medium	13.0	(10.8-15.5)	Medium	14.1	(11.2-17.7)
High	6.5	(3.8-10.8)	High	8.9	(5.4-14.2)
**Total (girls and boys)**	**15.4**	**(13.7-17.4)**	**Total (girls and boys)**	**15.4**	**(13.7-17.4)**

CI=confidence interval

**Table 2:** Prevalence of obesity (>97^th^ percentile) according to gender, age and socioeconomic status (n=1,799 girls, n=1,762 boys) Source: KiGGS Wave 2 (2014-2017)

Girls	%	(95% CI)	Boys	%	(95% CI)
**Girls (total)**	**5.5**	**(4.3-7.0)**	**Boys (total)**	**6.3**	**(4.9-8.0)**
**Age**			**Age**		
3-6 Years	3.2	(1.6-6.3)	3-6 Years	1.0	(0.4-2.5)
7-10 Years	4.7	(2.9-7.5)	7-10 Years	6.8	(4.2-11.0)
11-13 Years	6.5	(3.6-11.3)	11-13 Years	8.0	(4.8-13.0)
14-17 Years	7.7	(5.2-11.4)	14-17 Years	9.2	(6.2-13.4)
**Socioeconomic status**			**Socioeconomic status**		
Low	8.1	(4.7-13.7)	Low	11.4	(7.2-17.7)
Medium	4.7	(3.5-6.4)	Medium	5.2	(3.6-7.5)
High	2.0	(0.5-7.3)	High	2.6	(1.1-5.9)
**Total (girls and boys)**	**5.9**	**(5.0-7.0)**	**Total (girls and boys)**	**5.9**	**(5.0-7.0)**

CI=confidence interval
